# Supplementary material for: Streamlined copper defenses make Bordetella pertussis reliant on custom-made operon
Source: Commun Biol. 2021 Jan 8;4:46. doi: 10.1038/s42003-020-01580-2 (PMC7794356; doi:10.1038/s42003-020-01580-2)

Supplementary Figure S1. *B. pertussis* was grown in SS medium in the presence of 5 mM $CuSO_4$ (blue curves) or in standard conditions (orange curves), and 2 mM of fresh ascorbic acid (asco) was added to one of each pair of cultures (light orange and light blue) at the indicated time. The cultures represented by dark-color curves were not treated with additional ascorbic acid. The turbidity of the cultures was measured using an Elocheck device.

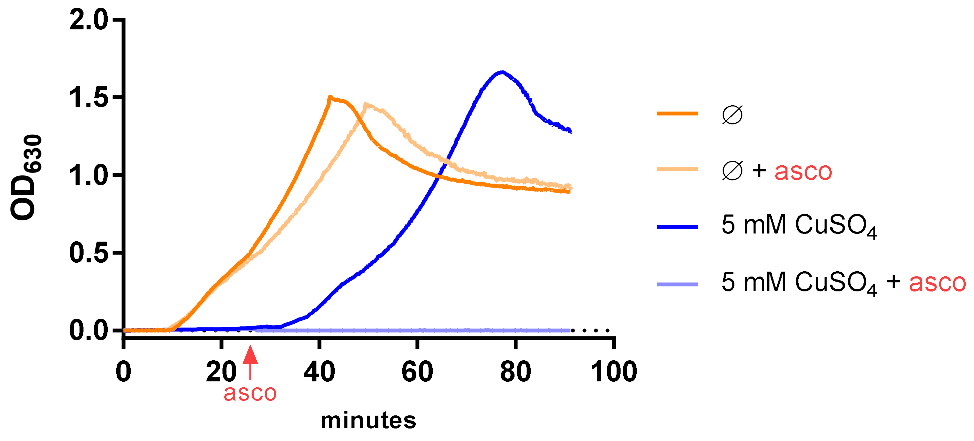

Supplementary Figure S2. RNAseq analyses of the *bp1727-bp1728-bp1729* locus showing its operonic structure. A screenshot of the mapping of RNAseq data shows that the three genes are co-transcribed and co-upregulated by copper and thus most likely form an operon. The data correspond to a *B. pertussis* culture subjected to 2 mM $CuSO_4$ for 30 min. Each horizontal green line below the three genes corresponds to one read in the sense of transcription of the operon. The preceding gene, *bp1726* is transcribed in the opposite direction, and thus a gap is visible between the *bp1726* transcripts and the *bp1727-bp1728-bp1729* transcripts.

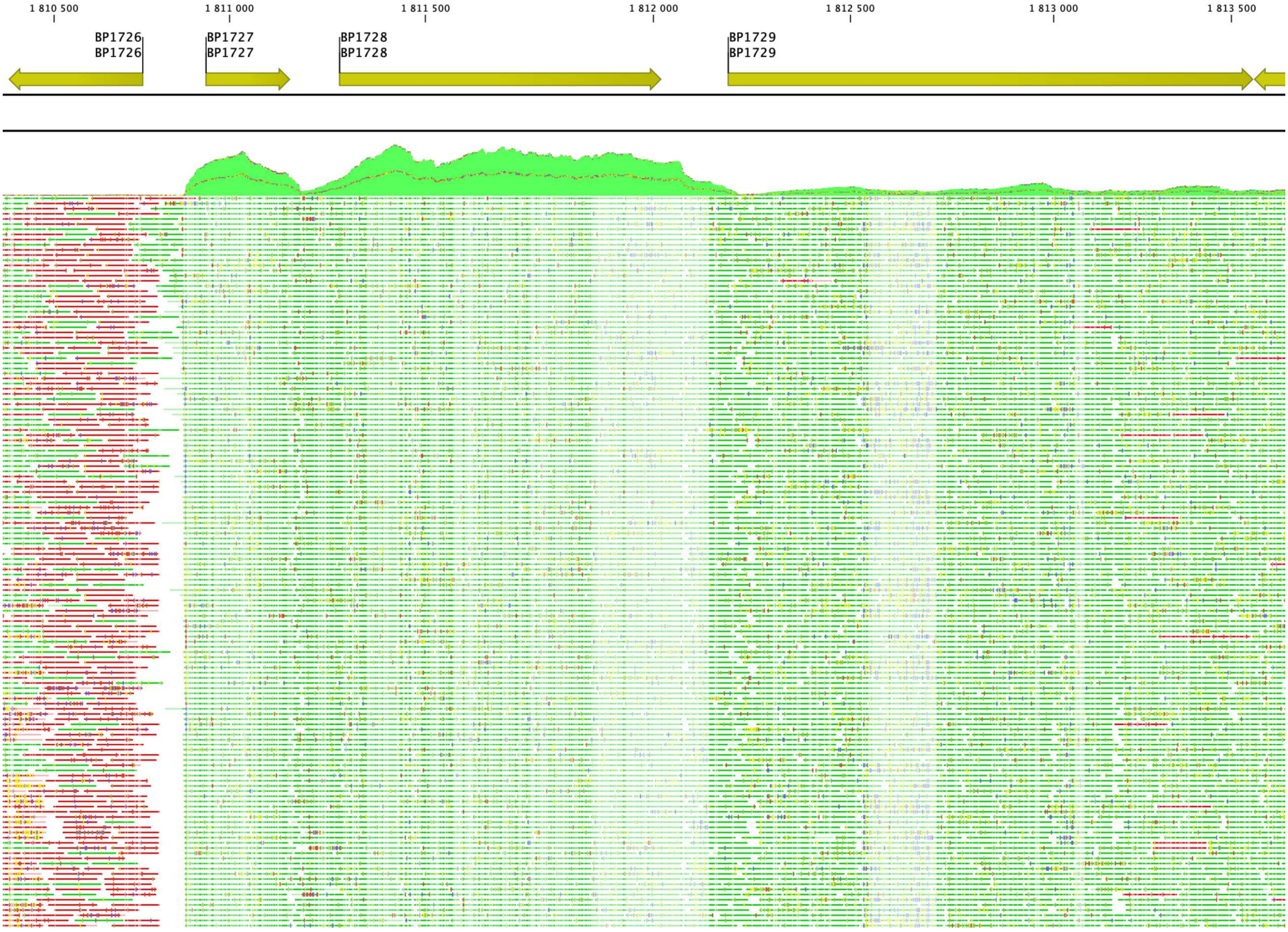

Supplementary Figure S3. Growth of *B. pertussis* mutants knocked-out for putative defense systems against copper toxicity. Growth yields of the parental strain, the Δ*bp1727* mutant (*copZ*), the Δ*bp2860* mutant (*copA*), the Δ*bp3314-bp3315-bp3316* mutant (*copI-pcoA- pcoB* operon), and Δ*bp0157-bp0158* mutant (*copRS*) grown for 24 h in standard SS medium or in medium supplemented with 2 mM $CuSO_4$ are shown. The cultures were inoculated at $0D_{600}$ values of 0.1, and after 24 h their OD_{600} values were measured. Three biological replicates were performed for each strain. Means and standard deviations are shown. Statistical analyses were performed using two-tailed Mann-Whitney tests (*, $p < 0.05$).

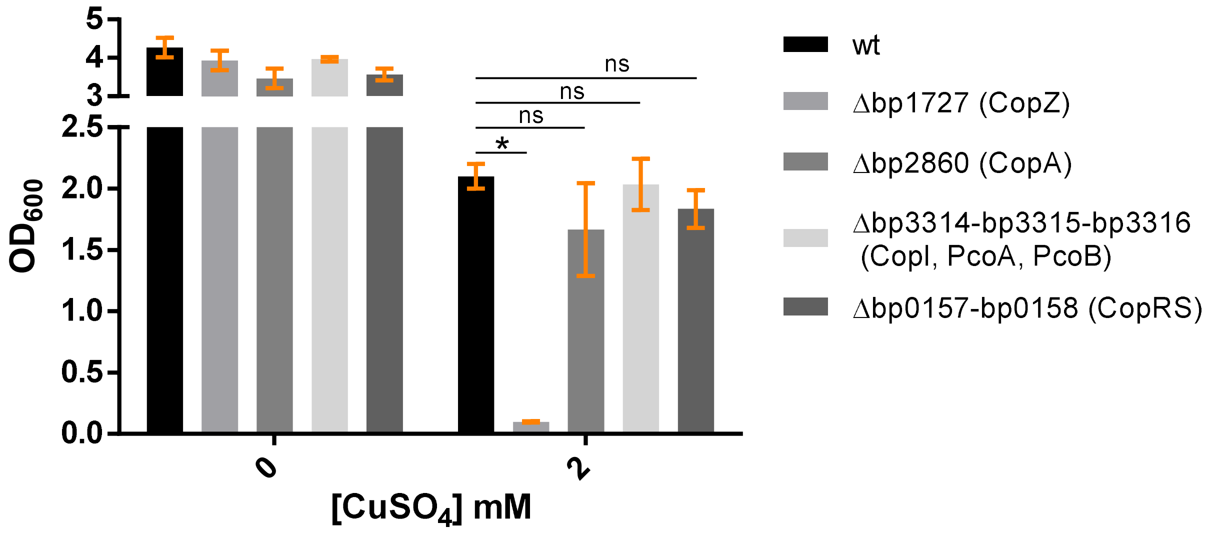

Supplementary Figure S4. ***copA* is interrupted *in B. pertussis***. The nucleotide sequences of relevant regions of the *copA* locus in *B. pertussis* (*bp2860*), *B. bronchiseptica* (*bb1180*) and *E. coli*, the position of the *IS481* insertion in *B. pertussis*, and the amino acid sequences of the first portions of CopA are shown. The nucleotides and amino acid residues that differ between *B. pertussis* and *B. bronchiseptica* are in grey letters. In *E. coli* (middle panel), the CueR binding boxes are within the promoter region (Yamamoto 2005, *Mol Microbiol* **56**, 215). Homologous motifs are present in the *copA* loci of *B. pertussis* and *B. bronchiseptica* (upper panels). In the latter, we mapped the transcription start site by 5' RACE (Supplementary Figure S5), which is denoted +1 in the figure. The -10 and -35 promoter boxes of the *Bordetella* locus thus appear to overlap the CueR binding site as in *E. coli*. Regarding translation, the initiation Met was probably mis-annotated in the *B. pertussis* genome, because sequence alignments of the N-terminal moieties of CopA from various species show that the CXXC copper-binding motif is usually located 12-25 residues after the initiation Met (lower panel). In the published *B. pertussis* annotation, a Met residue immediately preceding the CXXC motif was chosen for initiation, misleadingly suggesting a functional *copA* gene. However, the lack of a phenotype upon inactivation of *copA* strongly argues that the truncated gene no longer provides resistance against copper intoxication. Because the transposase gene in *IS481* is transcribed in the same direction and the transcript probably reads through *copA,* transcriptional activation of the latter was observed in the presence of copper (Fig. 2b and Supplementary Table S1).

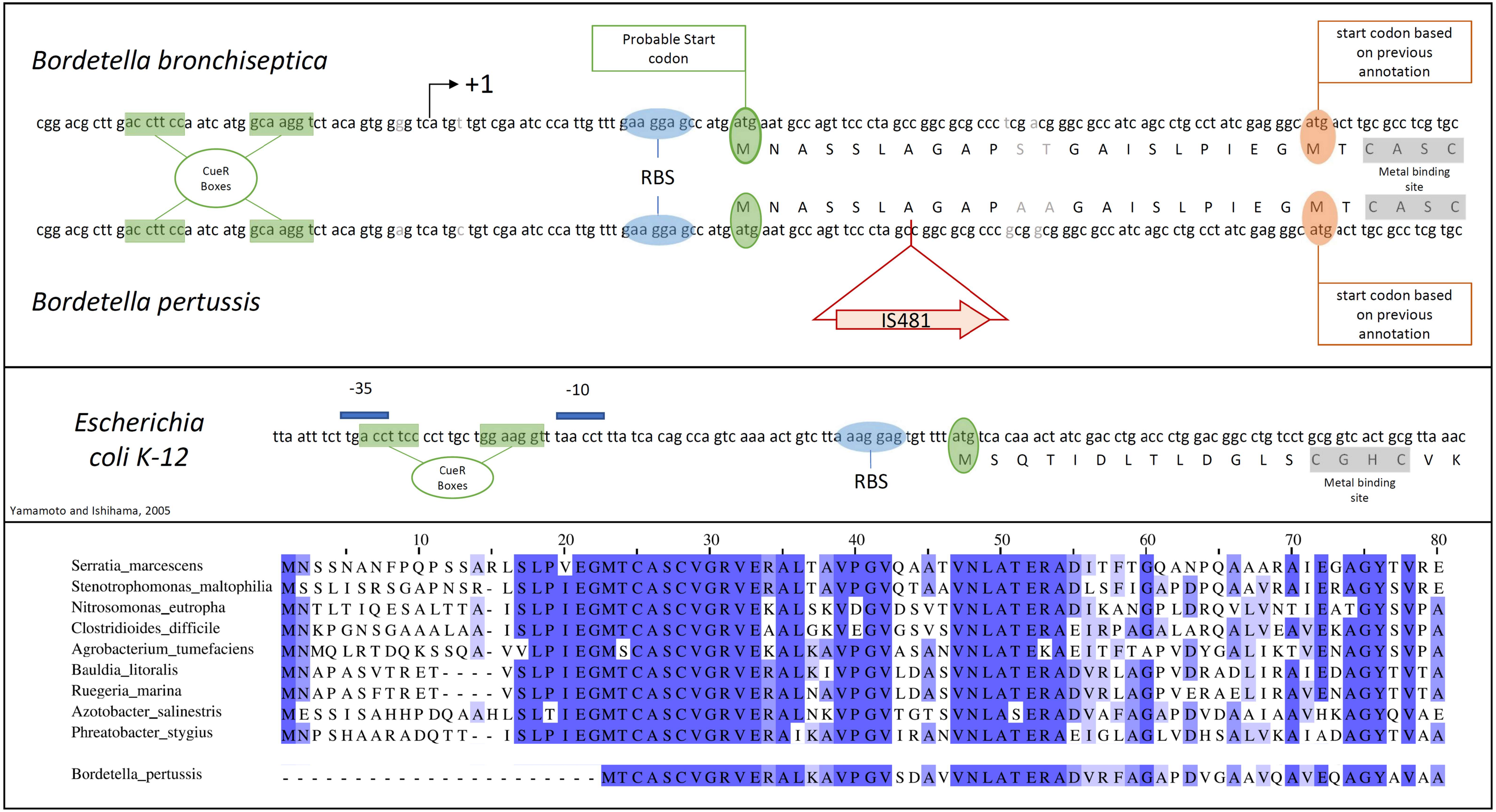

Supplementary Figure S5. **Identification of the transcription start sites of *bb1180* and *bp1727*.** Mapping of the transcriptional initiation sites was performed by rapid amplification of RNA 5' ends (5' RACE) using primers that anneal within *bb1180* (*copA*) in *B. bronchiseptica* and *bp1727* (*copZ*) in *B. pertussis*. The putative CueR boxes (in green) and -10 and -35 promoter boxes (in blue) are indicated. Those boxes were identified based on the sequences of the CueR boxes upstream of the *copA* gene in *E. coli* (Supplementary Figure S4). The -10 and -35 regions of the *bp1727* promoter are identical to those of the *copA* promoter in *E. coli* (Supplementary Figure S4). The transcriptional initiation sites of *bb1180* and *bp1727* are at the same distances from the CueR boxes in the two loci. The N-terminal regions of the two proteins are indicated in yellow, with their respective probable ribosome binding sites (RBS) circled and shaded in light blue. For *B. bronchiseptica* CopA, the annotated N terminus is most likely incorrect. The most probable initiation codon is circled and shaded in light green (see Supplementary Figure S4).

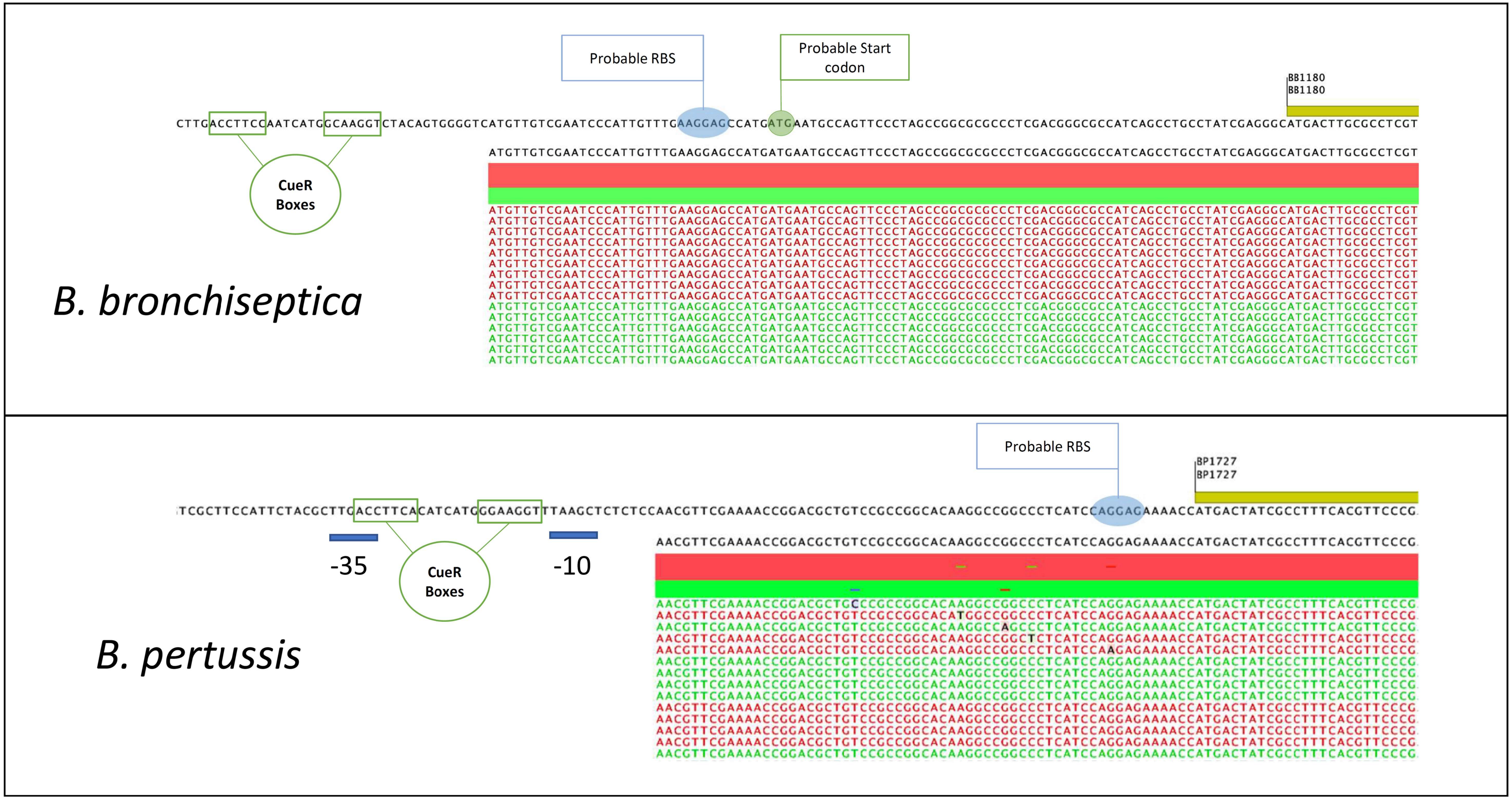

Supplementary Figure S6. Features of bacterial CopZ homologues. (**a**) Sequence alignments of various CopZ proteins were performed, showing the conserved CxHC motif. (**b**) The structure of CopZ from *Staphylococcus aureus* (pdb entry 6FF2) shows that the CxHC motif chelates copper, with a stoichiometry of 4 Cu ions (orange spheres) per CopZ dimer. The sulfhydryl groups of the Cys residues are in yellow, and the N and O atoms are in blue and red, respectively. The ratio of 2 Cu per CopZ monomer determined in this work is consistent with the structure presented here.

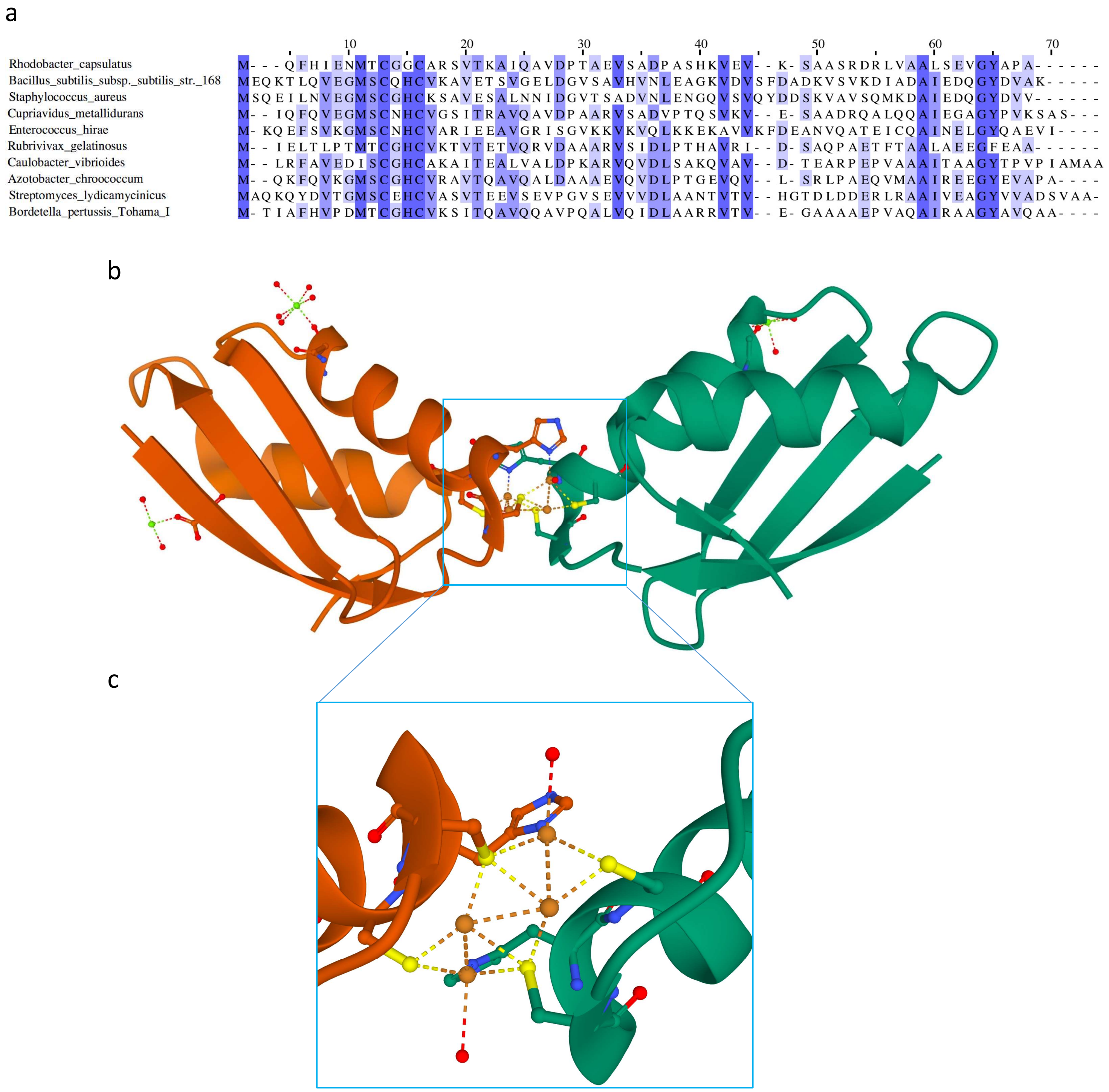

Supplementary Figure S7. Intracellular survival of *B. pertussis* in THP1 macrophages. The survival rates of the *copZ* KO mutant (Δ1727) and the *prxgrx-gorB* KO mutant (Δ1728-29) were compared with those of the parental strain and of the full deletion mutant (Δ1727-28-29). Three replicates were made for each strain. The means and standard deviations are shown (n = 4 to 6 replicates).

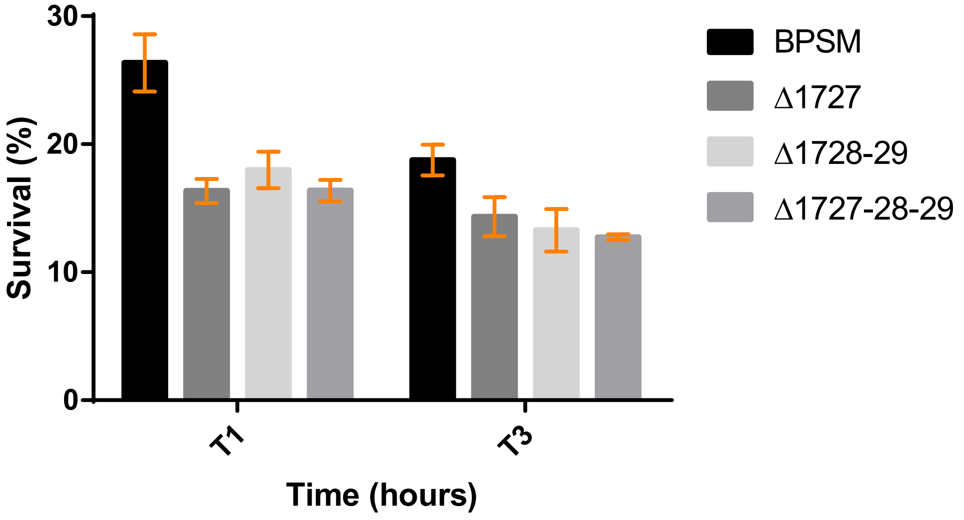

Supplementary Figure S8. Sensitivity of the transcriptional response to copper. qRT-PCR was performed on *prxgrx* after growing *B. pertussis* for 16 hours in SS medium containing the indicated concentrations of copper. Three technical replicates were performed at each concentration.

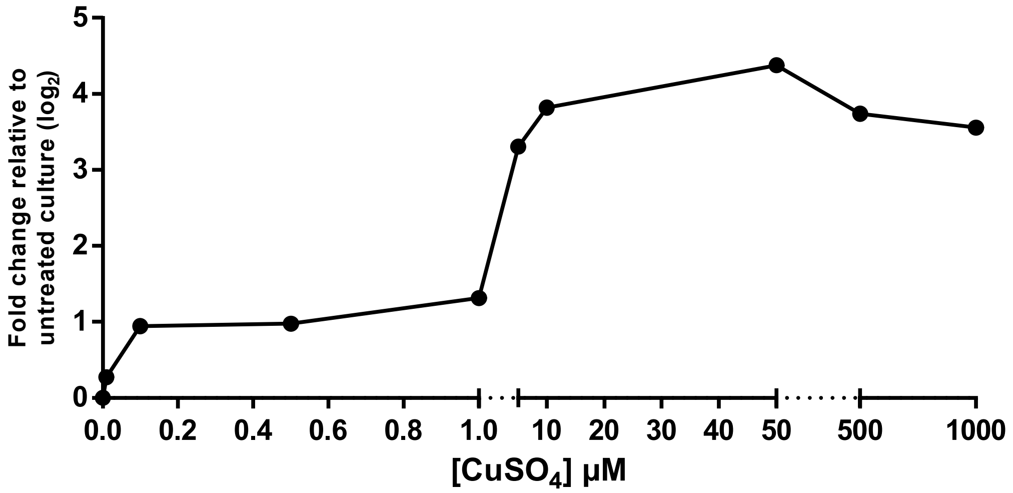

Supplementary Figure S9. Detection of the Cu transporter ATP7A in THP1 cells. (**a**) Representative fluorescence microscopy images of THP1 cells labelled for the ATP7A Cu transporter (in green), with the nuclei stained with DAPI (blue). The labelling was performed on non-differentiated monocytes (Ø), on monocytes differentiated for 24 hours into macrophages with PMA (PMA), with PMA and LPS (PMA + LPS), or with PMA before 2 hours of contact with *B. pertussis* (PMA + *Bp*). (**b**) Levels of green fluorescence (arbitrary units) of the cells labelled for ATP7A in the four conditions. Statistical analyses were performed using a non-parametric two-tailed Kruskal-Wallis test with Dunn's post-test (*****, p <0.0001*). The orange lines represent median values (n = 33 to 44 cells).

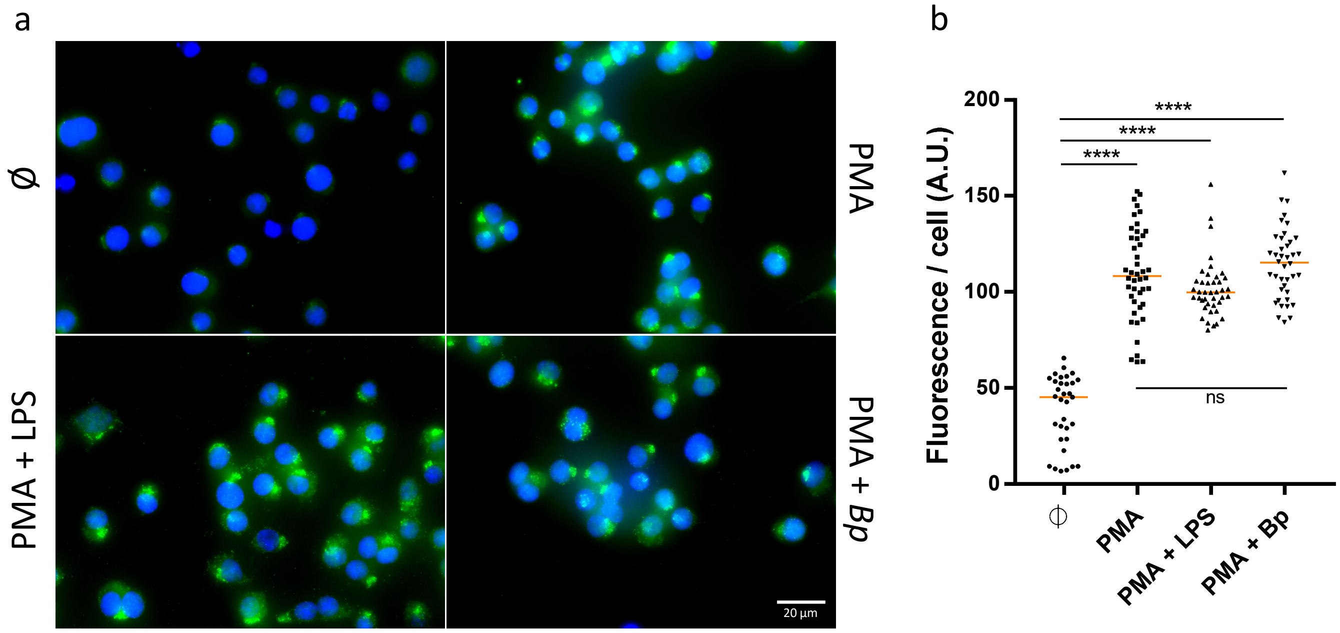

Supplementary Figure S10. Electrophoretic migration shift assay with OxyR. Recombinant OxyR (0, 2 or 20 mM) was incubated with the intergenic region between *cueR* and *copZ* (IGR 1) or with the intergenic region between *copZ* and *prxgrx* (IGR 2). The full ethidium-bromide-stained gel corresponding to the cropped gel shown in Figure 6 is presented here.

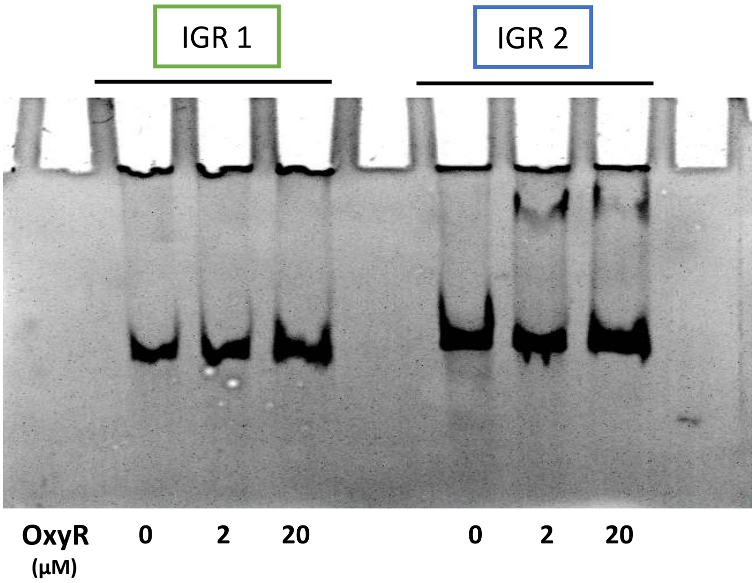

Supplementary Figure S11. Identification of putative OxyR boxes. Alignment of the *copZ-prxgrx* intergenic region of *B. pertussis* with those of other *Bordetella* and *Achromobacter* species. Based on the *E. coli* OxyR binding site consensus sequences (ATAGnnnnnnnCTAT) (Seo 2015, Cell Rep 12, 1289), at least two putative OxyR binding motifs were identified in the *copZ-prxgrx* intergenic region, but none was found in the *cueR-copZ* intergenic region. The spacing of 7 nucleotides between the two halves of the motif is conserved at both sites, and those motifs are well conserved among *Bordetellae* and *Achromobacter*. However, they both diverge by one nucleotide from the *E. coli* consensus, which might explain the rather weak binding of OxyR to IGR 2 as seen in Figure 6.

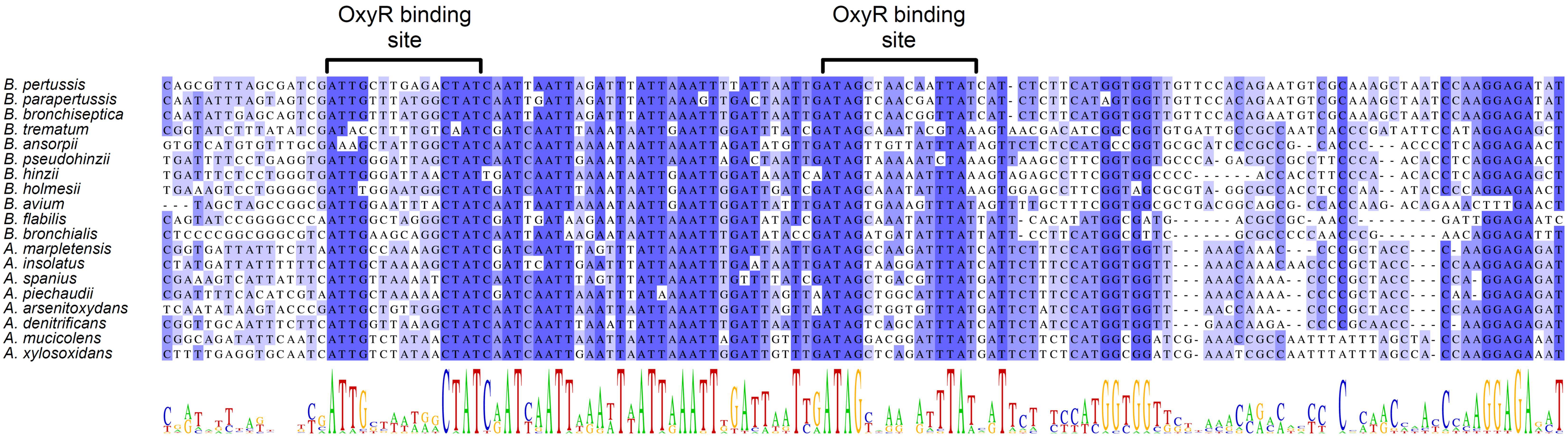

Supplement: Supplementary file 2 — Supplementary Information [file 42003_2020_1580_MOESM2_ESM.pdf]
