# Supplementary material for: Streamlined copper defenses make Bordetella pertussis reliant on custom-made operon
Source: Commun Biol. 2021 Jan 8;4:46. doi: 10.1038/s42003-020-01580-2 (PMC7794356; doi:10.1038/s42003-020-01580-2)
Supplement: Supplementary file 3 — Description of Additional Supplementary Files [file 42003_2020_1580_MOESM3_ESM.pdf]

Description of Additional Supplementary Files

Supplementary Data S1. Transcriptomic analyses of B. pertussis after a long exposure to copper. B. pertussis BPSM was grown in standard conditions or in the presence of 2 mM CuSO4, and RNA-Seq analyses were performed. The cultures were initiated at OD600 values of approximately 0.1, so that they reached OD600 of approximately 1.5 after 16 to 20 hours of growth. The RNAseq experiments were performed on two independent biological samples.

Supplementary Data S2. Transcriptomic analyses of B. pertussis after a brief exposure to copper. B. pertussis BPSM was grown in standard conditions to OD600 values of 1.5, and 2 mM CuSO4 was applied for 30 min. Control cultures were pursued untreated for the same time, and RNA-Seq analyses were performed. The RNAseq experiments were performed on two independent biological samples.

Supplementary Data S3. Transcriptomic analyses of B. bronchiseptica after a long exposure to copper. B. bronchiseptica RB50 was grown in standard conditions or in the presence of 2 mM CuSO4 for 10 to 12 h, and RNA-Seq analyses were performed. The cultures were initiated at OD600 values of 0.08, and the cultures were stopped when the OD600 reached 1.5 to 1.8. The RNAseq experiments were performed on two independent biological samples.

Supplementary Data S4. Proteomic analyses of B. pertussis. B. pertussis BPSM was grown in standard conditions or in the presence of 2 mM CuSO4. The cultures were initiated at OD600 values of 0.1, so that they reached OD600 of approximately 1.5 after 16 to 20 hours of growth. Cells were lysed, and soluble and insoluble proteins were separated by ultracentrifugation. They were analyzed by mass fingerprinting spectrometry after gel electrophoresis. Three independent biological samples were processed, and analysis of the pooled spectra from the soluble and insoluble fractions were performed for each of them. The p-values were calculated with a Student's T-test (confidence level: 95%), and to obtain q-values, the p-values were adjusted for multiple testing correction by following the Benjamini-Hochberg procedure (Q= 5%).

Supplementary Data S5. Proteomic analyses of B. bronchiseptica. B. bronchiseptica RB50 was grown in standard conditions or in the presence of 2 mM CuSO4. The cultures were initiated at OD600 values of 0.08, and the cultures were stopped after 10 to 12 hours when the OD600 reached 1.5 to 1.8. Cells were lysed, and soluble and insoluble proteins were separated by ultracentrifugation. They were analyzed by mass fingerprinting spectrometry after gel electrophoresis. Three independent biological samples were processed, and analysis of the pooled spectra from the soluble and insoluble fractions were performed for each of them. The p-values were calculated with a Student's T-test (confidence level: 95%), and to obtain q-values, the p-values were adjusted for multiple testing correction by following the Benjamini-Hochberg procedure (Q= 5%).

Supplementary Data S6. Genetic environment of prxgrx homologues in eubacteria. The 5 genes that precede and follow prxgrx in each species where it is present are indicated with their current annotations in the reference genomes. Note that when a gene was close to the extremity of the contig its distance to the adjacent gene could not be determined (nd).

Supplementary Data S7. Plasmids and oligonucleotides used in this study.

Supplementary Data S8. Data used to generate the charts and graphs.
