# Supplementary material for: Streamlined copper defenses make Bordetella pertussis reliant on custom-made operon
Source: Commun Biol. 2021 Jan 8;4:46. doi: 10.1038/s42003-020-01580-2 (PMC7794356; doi:10.1038/s42003-020-01580-2)
Supplement: Supplementary file 12 — Reporting Summary [file 42003_2020_1580_MOESM12_ESM.pdf]

natureresearch

Corresponding author(s): Jacob-Dubuisson Françoise

Last updated by author(s): Oct 27, 2020

Reporting Summary

Nature Research wishes to improve the reproducibility of the work that we publish. This form provides structure for consistency and transparency in reporting. For further information on Nature Research policies, see Authors & Referees and the Editorial Policy Checklist .

Statistics

For all statistical analyses, confirm that the following items are present in the figure legend, table legend, main text, or Methods section.

n/a	Confirmed	
☐	☒	The exact sample size (*n*) for each experimental group/condition, given as a discrete number and unit of measurement
☐	☒	A statement on whether measurements were taken from distinct samples or whether the same sample was measured repeatedly
☐	☒	The statistical test(s) used AND whether they are one- or two-sided<br>*Only common tests should be described solely by name; describe more complex techniques in the Methods section.*
☒	☐	A description of all covariates tested
☒	☐	A description of any assumptions or corrections, such as tests of normality and adjustment for multiple comparisons
☐	☒	A full description of the statistical parameters including central tendency (e.g. means) or other basic estimates (e.g. regression coefficient) AND variation (e.g. standard deviation) or associated estimates of uncertainty (e.g. confidence intervals)
☐	☒	For null hypothesis testing, the test statistic (e.g. *F*, *t*, *r*) with confidence intervals, effect sizes, degrees of freedom and *P* value noted<br>*Give P values as exact values whenever suitable.*
☒	☐	For Bayesian analysis, information on the choice of priors and Markov chain Monte Carlo settings
☒	☐	For hierarchical and complex designs, identification of the appropriate level for tests and full reporting of outcomes
☒	☐	Estimates of effect sizes (e.g. Cohen's *d*, Pearson's *r*), indicating how they were calculated

Our web collection on statistics for biologists contains articles on many of the points above.

Software and code

Policy information about availability of computer code

Data collection	To analyse the genetic environment of a gene of interest (prxgrx), the protein nr NCBI sequence database was searched for occurrence of Redoxin and Glutaredoxin domains signatures using hmmsearch from the HMMER package and the Redoxin.hmm and Glutaredoxin.hmm files downloaded from the Pfam database. A custom python script was used to select proteins carrying both domains. The sequence redundancy of the protein set was reduced using the cd-hit program form the CD-HIT Suite. The Pfam domains of the different proteins were defined using the Pfam Domain Search function of the CLC main Workbench software. The Taxonomy of the protein set was defined using the Bio.Entrez module of the BioPython package. The genetic environment of the genes coding for the different proteins was established using an in-house python script that parses the GenBank files of a local prokaryotic database composed of GenBank files from the BCT, ENV and CON divisions as well as RefSeq and WGS data.<br>For Imaging : Zen Software 2.3 SP1 FP1 (black)<br>For growth kinetics : Elocheck version 2.08<br>For qRT-PCR : LightCycler480 software release 1.5.0
Data analysis	For Imaging : Fiji (ImageJ) 1.52P, and Zen Software 2.3 SP1 FP1 (black)<br>For transcriptomics and 5'RACE: RockHopper version 2.03 and ClC Genomics Workbench 20<br>For proteomics : Proline version 2.0<br>To define patterns in DNA : Glam2 and Glam2Scan from MEME suite 5.1.0<br>For qRT-PCR : LightCycler480 software release 1.5.0<br>For Statistical analyses : Graphpad Prism 7.0

For manuscripts utilizing custom algorithms or software that are central to the research but not yet described in published literature, software must be made available to editors/reviewers. We strongly encourage code deposition in a community repository (e.g. GitHub). See the Nature Research guidelines for submitting code & software for further information.

Data

Policy information about availability of data

All manuscripts must include a data availability statement. This statement should provide the following information, where applicable:

- Accession codes, unique identifiers, or web links for publicly available datasets
- A list of figures that have associated raw data
- A description of any restrictions on data availability

To access transcriptomic data :
GEO accession GSE145049 go to https://www.ncbi.nlm.nih.gov/geo/query/acc.cgi?acc=GSE145049
The correspondence between the tables and the raw data is as follows:
Table S1 (GSM4305173 BPSM_Tris_Const Rep1; GSM4305174 BPSM_Tris_Const Rep2; GSM4305175 BPSM_Cu_Const Rep1; GSM4305176 BPSM_Cu_Const Rep2)
Table S2 (GSM4305169 BPSM_Tris_Shock Rep1; GSM4305170 BPSM_Tris_Shock Rep2; GSM4305171 BPSM_Cu_Shock Rep1; GSM4305172 BPSM_Cu_Shock Rep2)
Table S3 (GSM4305177 BbRB50_Tris_Const Rep1; GSM4305178 BbRB50_Tris_Const Rep2; GSM4305179 BbRB50_Cu_Const Rep1; GSM4305180 BbRB50_Cu_Const Rep2)

To access proteomic data :
- Project Name: Streamlining of defenses against copper makes host-restricted pathogen reliant on custom-made operon
- Project accession: PXD020900
- Project DOI: 10.6019/PXD020900

Field-specific reporting

Please select the one below that is the best fit for your research. If you are not sure, read the appropriate sections before making your selection.

☒ Life sciences ☐ Behavioural & social sciences ☐ Ecological, evolutionary & environmental sciences

For a reference copy of the document with all sections, see nature.com/documents/nr-reporting-summary-flat.pdf

Life sciences study design

All studies must disclose on these points even when the disclosure is negative.

Sample size	All in vitro experiments and their statistical analyses were performed using at least 3 biological replicates. For the animal colonization experiment there were 4 mice per time point for wild type bacteria (as the colonisation kinetics of this strain is well known) and 5 mice per time point for the mutant strain. The first time point was performed with only 3 mice per group to check for similar bacterial loads in mice at the beginning of the experiment.
Data exclusions	Two mice were excluded from the experiments: one died upon anesthesis, and the other was sacrificed because of another infection. The values obtained with the latter mouse were discarded.
Replication	In addition to biological and technical replicates, all experiments (except animal colonization) were repeated at least 2 times independently.
Randomization	Randomization was not necessary for the in vitro experiments. For animal experiments, mice were randomly assigned to two groups.
Blinding	Blinding was not necessary in this study.

Reporting for specific materials, systems and methods

We require information from authors about some types of materials, experimental systems and methods used in many studies. Here, indicate whether each material, system or method listed is relevant to your study. If you are not sure if a list item applies to your research, read the appropriate section before selecting a response.

Materials & experimental systems

n/a	Involved in the study	
☒	☐	Antibodies
☐	☒	Eukaryotic cell lines
☒	☐	Palaeontology
☐	☒	Animals and other organisms
☒	☐	Human research participants
☒	☐	Clinical data

Methods

n/a	Involved in the study	
☒	☐	ChIP-seq
☒	☐	Flow cytometry
☒	☐	MRI-based neuroimaging

Eukaryotic cell lines

Policy information about cell lines

Cell line source(s)	THP1 from ATCC cat# TIB-202
Authentication	Cell line was not authenticated.
Mycoplasma contamination	The cells are free of mycoplasma. This was tested with the Mycoalert kit.
Commonly misidentified lines (See ICLAC register)	n/a

Animals and other organisms

Policy information about studies involving animals; ARRIVE guidelines recommended for reporting animal research

Laboratory animals	Female, 6-weeks-old JAX ™ BALB/ cByJ mice
Wild animals	n/a
Field-collected samples	n/a
Ethics oversight	All the experiments were carried out in accordance with the guidelines of the French Ministry of Research regarding animal experiments, and the protocols were approved by the Ethical Committees of the Region Nord Pas de Calais and the Ministry of Research (agreement number APAFIS#9107±201603311654342 V3).

Note that full information on the approval of the study protocol must also be provided in the manuscript.
